# A Systematic Review and Meta-Analysis of the Efficacy and Safety of Sodium-Glucose Cotransporter-2 Inhibitor in Patients Using Left Ventricular Assist Devices

**DOI:** 10.3390/jcm13237418

**Published:** 2024-12-05

**Authors:** Elfatih A. Hasabo, Burce Isik, Ammar Elgadi, Mohammed Mahmmoud Fadelallah Eljack, Magdi S. Yacoub, Hesham Elzomor, Sherif Sultan, Kadir Caliskan, Osama Soliman

**Affiliations:** 1 Discipline of Medicine, School of Medicine, College of Medicine Nursing and Health Sciences, University of Galway, H91 TK33 Galway, Ireland; elfatih.ahmed.hasabo@gmail.com (E.A.H.); magdisyacoub@gmail.com (M.S.Y.);; 2 Discipline of Cardiology, Saolta Healthcare Group, Galway University Hospital, Health Service Executive, H91 YR71 Galway, Ireland; 3 CORRIB-CURAM-VASCULAR Group, University of Galway, H91 YR71 Galway, Ireland; 4 School of Medicine, University of Limerick, V94 T9PX Limerick, Ireland; 5 Faculty of Medicine, University of Khartoum, Khartoum 11111, Sudan; 6 Faculty of Medicine, University of Bakht Alruda, Ad Duwaym 11112, Sudan; 7 Department of Vascular and Endovascular Surgery, Western Vascular Institute, University College Hospital, H91 YR71 Galway, Ireland; 8 Thoraxcenter, Department of Cardiology, Cardiovascular Institute, Erasmus MC University Medical Center, 3015 GD Rotterdam, The Netherlands; k.caliskan@erasmusmc.nl; 9 Euro Heart Foundation, 3071 Rotterdam, The Netherlands

**Keywords:** sodium-glucose cotransporter-2 inhibitors, ventricular assist device, systematic review, meta-analysis, efficacy, safety

## Abstract

**Background:** Sodium-glucose cotransporter-2 inhibitors (SGLT2-i) have been shown to reduce risks of clinical events in patients with heart failure (HF). However, data on the use of SGLT2-i in patients with left ventricular assist devices (LVADs) are scarce. We thought to assess the efficacy and safety of SGLT2-i in patients with LVADs. **Methods:** A systematic search was conducted in PubMed, Scopus, Web of Science, Embase, and Cochrane from inception to November 2024. We used all relevant words for “SGLT2-i” and “LVAD” to search in databases, and we included studies and published abstracts in peer-reviewed journals of studies that assessed SGLT2-i in patients with LVAD. **Results:** Four studies and seven abstracts totaling 228 patients using SGLT2-i were included. Empagliflozin, Dapagliflozin, and Canagliflozin were the used SGLT2-i across the included studies. Pooled analysis showed that SGLT2-i significantly improved ejection fraction (EF) (Mean= 4.2, 95% CI [1.22, 7.19]) and hemoglobin A1c (HbA1c) (Mean = −0.44, 95% CI [−0.79, −0.09]) from baseline. However, no significant changes in B-type natriuretic peptide (BNP), or glomerular filtration rate (GFR) were noticed. Other outcomes of interest not included in the meta-analysis did not show significant changes, such as cardiac index (CI), left ventricular end-systolic diameter (LVESD), left ventricular end-diastolic diameter (LVEDD), mean arterial pressure (MAP), or mean pulmonary artery pressure (MPAP). The pooled percentage of people with driveline infection was 9%, 95% CI (3, 19). **Conclusions:** SGLT2-i effectively improves EF and HbA1c in patients using LVAD. Further adequately powered randomized studies are warranted to ascertain its clinical efficacy and safety in that unique population.

## 1. Introduction

Sodium-glucose cotransporter-2 inhibitors (SGLT2-i) are becoming increasingly important in the management of heart failure (HF) due to their therapeutic effects in patients with or without diabetes. Hence, there is an abundant amount of research exploring the relationship between SGLT2-i and their use in patients with chronic HF. A DAPA-HF trial showed that patients with HF and reduced ejection fraction, irrespective of their Type 2 Diabetes Mellitus (T2DM) status, had less hospitalization due to acute decompensated HF and death from cardiovascular events, which suggests an unexplored mechanism of action of SGLT2-i other than glycosuria and diuresis [1]. The EMPULSE trial reported the safe and effective use of SGLT2-i in acute decompensated HF, resulting in reduced hospitalization from cardiovascular events [2]. The EMPEROR trial showed reduced HF-related hospitalization in patients with preserved ejection fraction (HFpEF), making SGLT2-i a potential candidate for early and safe treatment in the HFpEF population [3]. For the preceding reasons, SGLT2-i can benefit various patient populations as an early intervention.

LVADs are widely used in the treatment of advanced heart failure, as the number of patients with advanced heart failure is increasing worldwide [4]. However, there is a lack of data on the use of SGLT2-i in the population supported by left ventricular assist devices (LVADs) and the impact of SGLT2-i on LVADs and patient outcomes. There are few studies conducted in the LVAD population treated with SGLT2-i, but all of these studies have a small number of cohorts. A study of the use of SGLT2-i in 31 patients with LVADs as BTT has concluded that SGLT2-i is safely tolerated in this patient population [5]. Another study of the outcomes of SGLT2-i in 20 diabetic patients who were wearing LVADs has reported that SGLT2-i are safe to use with no significant effect on LVAD pump function and mean arterial pressure (MAP), with a transient decrease in renal function [6]. Cagliostro et al. evaluated the safety of SGLT2-i in the LVAD population with T2DM and found non-SGLT2-i specific adverse events, such as acute kidney injury, urinary tract infections, and limb amputations [7]. Further research in larger cohorts of patients treated with LVADs and SGLT2-i is warranted to strengthen these findings.

The therapeutic effects of LVADs combined with the diuretic effect of SGLT2-i may further reduce pulmonary artery pressure and improve right ventricle and kidney function [5]. An EMBRACE-HF trial showed the pulmonary artery pressure reduced by the effects of SGLT2-i in patients with HFrEF [8]. However, this cohort did not include patients supported by LVADs. A major complication after LVAD transplantation is the management of high blood pressure [4]. These effects cannot be explained only by SGLT2-i-induced diuresis and glycosuria [9]. Chen et al. suggested that SGLT2-i plays a role in preventing the cascade of multiple molecular events underlying the pathogenesis of heart failure [9]. Further studies are needed to explore the mechanism behind these effects of SGLT2-i.

This study aims to summarize the existing knowledge on the use of SGLT2-i in patients with heart failure wearing LVADs, its safety for use with LVADs, and its effects on primary and secondary patient outcomes. This study identifies the knowledge gaps in the use of SGLT2-i in patients supported by ventricular assist devices and their combined effects on heart failure outcomes and potential cardiac recovery.

## 2. Methods and Materials

We conducted a systematic review and meta-analysis of the efficacy and safety of SGLT2-i among patients using LVADs according to the Cochrane Handbook for systematic reviews of interventions [10]. This systematic review was reported using the preferred reporting items for systematic review and meta-analysis (PRISMA statement) [11]. We started the execution of this project by performing data extraction before protocol registration; hence, this review was not eligible for PROSPERO protocol registration. This systematic review included any patient using a LVAD and any SGLT2-i.

### 2.1. Search Strategy

We searched PubMed, Scopus, Web of Science, Embase and Cochrane databases by using the following keywords (SGLT2 OR “SGLT2 inhibitor” OR “SGLT-2 inhibitor” OR “sodium glucose cotransporter 2 Inhibitor” OR “Sodium Glucose Transporter 2 Inhibitors” OR “sodium glucose co-transporter 2” OR “ sodium glucose co-transporter 2 inhibitor” OR “ sodium glucose cotransporter 2” OR “SGLT-2i” OR gliflozin OR Gliflozins OR canagliflozin OR empagliflozin OR dapagliflozin OR Ipragliflozin OR tofogliflozin OR luseogliflozin OR ertugliflozin OR sotagliflozin OR remogliflozin OR Invokana OR Farxiga OR Jardiance OR Suglat OR Apleway OR Deberza) AND (“left ventricular assist device” OR lvad OR heartmate OR heartware OR “heart pump” OR jarvik-2000 OR thoratec OR “ventricular assist device” OR vad OR “Mechanical Circulatory Support” OR mcs OR “biventricular assist device” OR bivad OR Heart Assist Device OR novacor OR “left ventricular assist system” OR LVAS OR RVAD OR right ventricular assist device) from inception to 18 November 2024. Details for the search used in the different databases are found in Supplementary Table S1.

### 2.2. Eligibility Criteria and Study Selection

In this systematic review, we included all studies that met the following criteria:

1. Randomized control trial (RCT), case reports, cohort studies, case–control studies, conference abstracts published in peer-reviewed journals, or observational studies that included patients with LVADs using SGLT2-i.
2. Studies in the English language.

Different exclusion criteria were set for this systematic review, as follows:

1. Review articles, opinion papers, systematic reviews, or study protocols.
2. Published theses.
3. Studies with languages other than English.
4. In vitro or animal studies.
5. Duplicate studies and articles not published yet.

Independently, the reviewers screened all the studies retrieved from databases during the title and abstract screening, and the remaining studies were entered in the full-text screening. Then, full-text screening was performed independently to obtain the final included studies.

### 2.3. Data Extraction

The authors extracted the following data from the included studies:

1. Baseline information and summary of included studies.
2. Efficacy outcomes of SGLT2-i in patients using LVADs.
3. Safety of SGLT2-i in patients using LVADs: Driveline infection, diabetic ketoacidosis (DKA), and acute kidney injury (AKI).

### 2.4. Quality Assessment

We assessed the quality using the Newcastle–Ottawa Scale (NOS) and adapted the NOS [12]. For the single-arm studies, we assessed the quality using the adapted NOS. The total score was 6 after excluding the questions of “selection of the non-exposed cohort” and the comparability question “Comparability of cohorts based on the design or analysis”.

### 2.5. Data Analysis

Data from the included studies were extracted and presented as tables and figures. Meta-analysis was conducted using R with the libraries dmetar, meta, and tidyverse. Pooled continuous data were calculated using the function “metamean” presented as mean and 95% CI. Pooled categorical data were computed using the function “metaprop” presented as proportion and 95% confidence interval (CI). Missing mean and standard deviation (SD) were calculated using the median, standard error (SE), or 95% CI, according to Altman [13]. The I-square (I2) test was used to assess the heterogeneity. The chi-square test was considered significant with a *p* value > 0.1, indicating no heterogeneity.

## 3. Results

### 3.1. Literature Search

**Figure 1:**
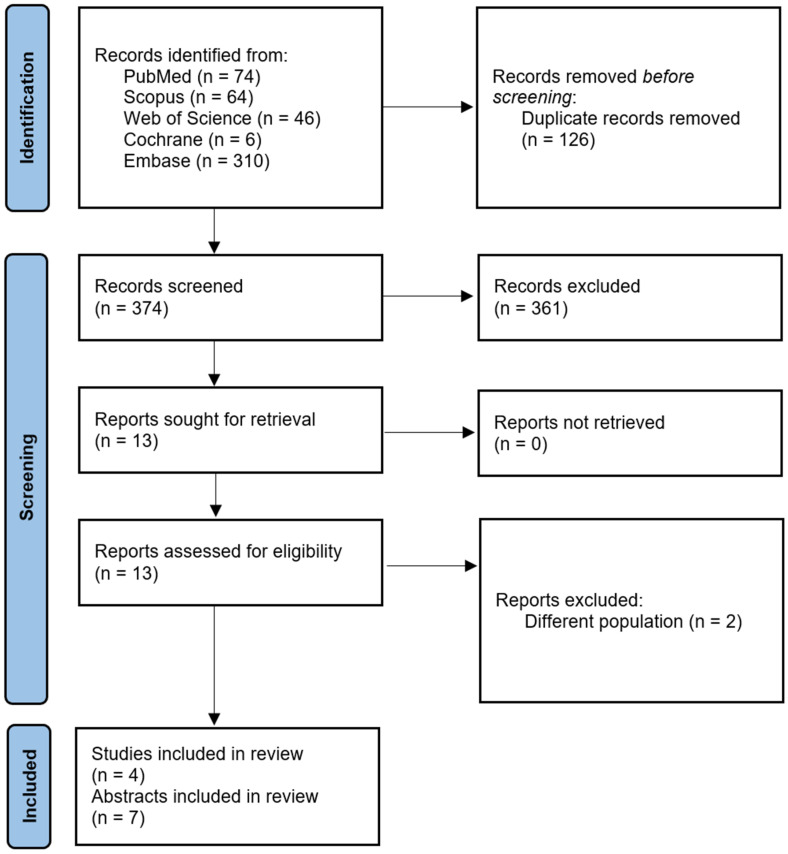
PRISMA Flow Diagram.

The PRISMA flow diagram in Figure 1 describes the literature search conducted in this study. Five hundred potentially relevant publications were identified after searching PubMed, SCOPUS, Web of Science, Embase, and Cochrane. After removing duplicates, 374 publications remained. After title and abstract screening, 13 full-text articles were assessed for eligibility. Two of these publications were excluded. Finally, the qualitative and quantitative analysis included four studies and seven published abstracts that met our inclusion criteria [5,6,7,14,15,16,17,18,19,20,21].

### 3.2. Summary and Baseline Data of Included Studies

A total of 228 LVAD patients using SGLT2-i were included in our meta-analysis. Empagliflozin, Dapagliflozin, and Canagliflozin were the SGLT2-i used in the included studies. The descriptive baseline data and types of SGLT2-i used among participants are found in Table 1 and Table 2, and a summary of the included studies is shown in Table 3.

### 3.3. Quality Assessment of Included Studies

Quality assessment using the adapted NOS and Cochrane Risk of Bias tool showed a low risk of bias among the included studies. Further details about the quality assessment are found in Supplementary Table S2.

### 3.4. Efficacy of SGLT2-i

1. Change from baseline in ejection fraction (EF)

**Figure 2:**
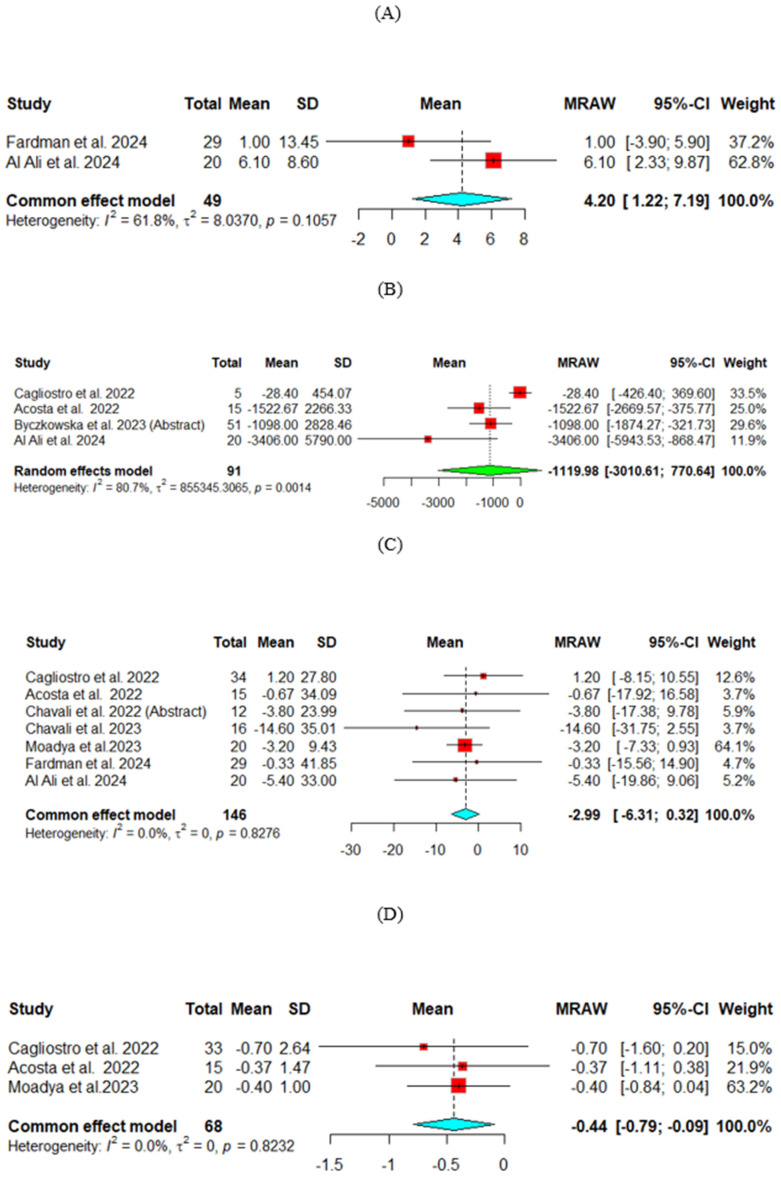
Efficacy outcomes of SGLT2-i for change from baseline in (**A**) Ejection fraction, (**B**) B-Type Natriuretic Peptide, (**C**) Glomerular filtration rate, (**D**) Hemoglobin A1c.

Two studies assessed the change in EF, and the pooled analysis showed significant improvement for EF (Mean = 4.2, 95% CI [1.22, 7.19]). (Figure 2A)

2. Change from baseline in B-Type Natriuretic Peptide (BNP) (pg/mL)

The change from baseline in BNP was compared across four studies. The random effect model was used for analysis. The heterogeneity across the studies was high (*p* = 0.001, I^2^ = 81%). The results did not show a significant decrease in BNP (Mean = −1119.98, 95% CI [−3010.61, 770.64]). (Figure 2B)

3. Change from baseline in glomerular filtration rate (GFR) (mL/min/1.73 m^2^)

The change from baseline in GFR was compared across seven studies. The common effect model was used for analysis. The results showed no significant improvement (Mean = −2.99, 95% CI [−6.31, 0.32]) (Figure 2C)

4. Change from baseline in hemoglobin A1c (HbA1c)

The change from baseline HbA1c was compared across three studies. The common effect model was used for analysis. The results showed a significant decrease from baseline in Hb1Ac (Mean = −0.44, 95% CI [−0.79, −0.09]), suggesting that SGLT2-i treatment in the LVAD population improves HbA1c effectively. (Figure 2D)

5. Outcomes not included in the meta-analysis:

Due to a lack of data in this systematic review, several outcomes were not included in the pooled meta-analysis. Echocardiography parameters and outcomes of interest such as cardiac index (CIn), left ventricular end-systolic diameter (LVESD), left ventricular end-diastolic diameter (LVEDD), mean arterial pressure (MAP), mean pulmonary artery pressure (MPAP), right atrial pressure (RAP), or systemic vascular resistance (SVR) did not show significant change after using SGLT2-i among participants. However, significant improvement in New York Heart Association (NYHA) class was noticed after 3 months and 1 year after initiating SGLT2-i [18], and for quality of life when assessed using the Kansas City Cardiomyopathy Questionnaire (KCCQ) [20]. Further details are available in Table 4 and Supplementary Table S3.

### 3.5. Safety of SGLT2-i

1. Driveline infection

**Figure 3:**
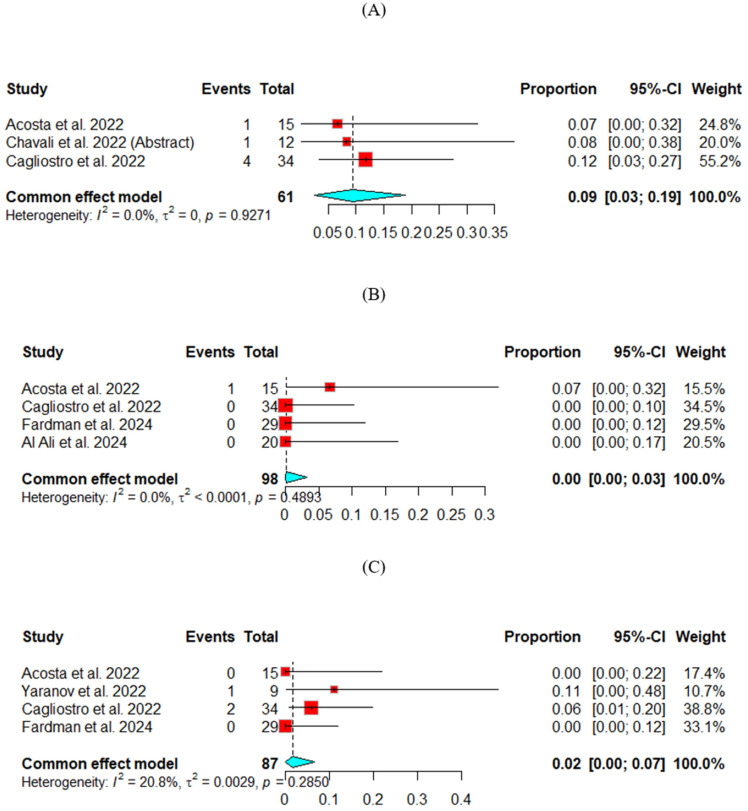
Safety outcomes for SGLT2-i: (**A**) Driveline infection, (**B**) Diabetic ketoacidosis, (**C**) Acute kidney injury.

The driveline infection rate was measured across three studies. The fixed effect model was used for analysis. The pooled percentage of people with driveline infection was 9%, 95% CI (3, 19). (Figure 3A)

2. Diabetic ketoacidosis (DKA)

The DKA rate was measured across two studies. The fixed effect model was used for analysis. The pooled percentage of people with DKA was 0%, 95% CI (0, 3). (Figure 3B)

3. Acute kidney injury (AKI)

The AKI rate was measured across four studies. The fixed effect model was used for analysis. The pooled percentage of people with AKI was 2%, 95% CI (0, 7). (Figure 3C)

## 4. Discussion

This is the first systematic review and meta-analysis investigating the use of SGLT2-i in patients using LVADs. Our meta-analysis showed that SGLT2-i improved EF and HbA1c levels among patients with LVADs. However, no significant change was noticed in BNP and GFR levels. Furthermore, SGLT2-i showed a low incidence of driveline infection and acute kidney injury. Also, NYHA class and quality of life using KCCQ showed improvement after using SGLT2-i in this systematic review.

SGLT2-i is not widely used in patients using LVADs as a destination therapy or bridge-to-transplantation (BTT) [5,6]. However, patients supported with LVADs can still experience symptoms of HF, which SGLT2-i can address. Therefore, the effects of SGLT2-i in this patient population require more attention.

SGLT2 inhibitors have a positive effect in improving mortality and outcomes [22]. Additionally, SGLT2-i showed a reduction in atrial fibrillation among patients [23].

SGLT2 inhibitors have shown to be a game-changing option for individuals with type 2 diabetes. In this study, we investigated the efficacy and safety of SGLT-2 inhibitors in patients using LVADs. Our findings indicate that SGLT-2 inhibitors can significantly improve EF and HbA1c in LVAD patients, but pooled analysis showed no significant change in BNP and eGFR.

These oral drugs work by inhibiting reabsorption in proximal tubules, leading to increased urinary excretion of glucose, improving blood pressure, renal function, and cardiovascular outcomes. The ability of these inhibitors to enhance natriuresis and osmotic diuresis results in a reduction in arterial stiffness, afterload, and subendocardial blood flow, ultimately leading to a decrease in left ventricular end-diastolic volume and a reduction in mortality and frequency of admission [24,25]. Notably, even individuals with LVAD can benefit from the natriuretic effect of these inhibitors. These patients may have improved functional capacity and prognosis but still present the clinical characteristics of heart failure and require continuous diuretic therapy. These inhibitors have been shown to potentiate diuretic therapy, as demonstrated by the findings of Colvin et al., 2021 [26]. Moreover, Zinman et al. found that the placebo group required a higher diuretic dose, highlighting the potential benefits of these inhibitors [25].

One of the most important parameters in clinical practice for the assessment of heart failure is the measurement of left ventricular systolic function, which is assessed mainly via EF [27]. Our pooled analysis showed significant improvement in EF, and it was assessed in two included studies [20,21], which highlights the need for large RCTs to confirm the effect of SGLT2-i on EF. A previous systematic review of SGLT2-i confirmed this finding and revealed a significant improvement in EF and reversed cardiac remodeling in patients with HF [28].

The level of BNP has long been considered a predictor for heart failure severity, health status, and hospitalization risk or cardiovascular death. Higher levels of NT-proBNP reflect a more decompensated hemodynamic profile, higher filling pressure, and greater risk of progressive cardiac remodeling, hospitalization, and death [29]. Conversely, reducing NT-proBNP levels following heart failure treatment predicts improved outcomes and health status. Our study found no significant change in the pooled BNP, which is consistent with the findings of the DEFINE-HF Trial for dapagliflozin and CANVAS for canagliflozin, while the DAPA-HF trial and EMPEROR-Reduced Trial James reported significant reductions in pro-BNP from baseline to the end of follow-up, albeit with low cardiovascular risk outcomes [1,29,30,31]. All included studies in the meta-analysis showed a significant reduction in the BNP, except Cagliostro et al., 2022 [7] did not show any significant change, which can be explained by using different types of SGLT2-i. Another post hoc study of the EMPEROR-Preserved Trial showed that Empagliflozin led to decreased NT-proBNP levels in comparison with the placebo group [32].

The inadequacy of cardiac output leading to congestion and secondary pulmonary hypertension continues to be one of the primary endpoints of heart failure, often resulting in hospitalization and mortality. Left ventricular assisted devices have been employed as a potential solution for this hemodynamic abnormality, as they increase cardiac output, decrease pulmonary wedge capillary pressure, and reduce pulmonary vascular resistance [33]. While previous studies conducted by Omar et al. and Kirschbaum et al. reported significant reductions in systolic pulmonary artery pressure and MPAP, our study revealed no significant changes in MPAP upon introducing SGLT2-i drugs [21,33,34].

Sodium-glucose cotransporter-2 inhibitors have been associated with a reduction in intraglomerular pressure and an increase in GFR, secondary to tubule-glomerular feedback in response to the increased salt delivery via the inhibition of sodium transport proximally. Furthermore, an increase in sodium chloride delivery to the macula densa may suppress the renin-angiotensin-aldosterone system and provide renoprotection, given that the plasma level of this system is already high [33,34]. In our study, no significant change was observed in the level of GFR, while the EMPEROR-reduced and DAPA-HF trials reported significant reductions in GFR in their studies [1,29]. A possible explanation is that the results of GFR may be affected by the existing use of RAAB. Nevertheless, these changes tend to reverse after stopping treatment. The DECLARE-TIMI and EMPA-REG OUTCOMES trials revealed that the use of dapagliflozin resulted in a significant reduction in the urinary albumin–creatinine ratio [25,35]. This finding suggests that SGLT2 inhibitors may be a viable option for the treatment of albuminuria in patients with type 2 diabetes.

Patients with heart failure often exhibit low cardiac output, leading to an increase in the levels of circulating catecholamines and an increase in sympathetic activity. Consequently, this increase leads to inhibiting pancreatic insulin secretion and stimulating hepatic gluconeogenesis and glycogenolysis, resulting in hypoglycemia and an increase in insulin requirement [36]. This phenomenon leads to a reduction in insulin sensitivity in patients with heart failure by 58%, and as estimated by Framingham, diabetes mellitus multiplies the risk of heart failure by eight times, and this risk is primarily dependent on the level of glycemic control, precisely the level of HbA1c [36].

These consequences of low cardiac output and pancreatic congestion should be reversed with the use of LVADs, leading to better HbA1c control, as demonstrated by our study and those of Guglin et al. and Chokshi et al. [36,37].

The use of SGLT2-i drugs, on the other hand, has been associated with an increased risk of diabetic ketoacidosis. One possible explanation is that SGLT2-i acts as an insulin-independent agent, lowering the required insulin dose and causing ketosis to worsen. This is further exacerbated by increased sodium reabsorption within renal tubules, which interferes with ketone clearance and increases circulating ketones and, ultimately, diabetic ketoacidosis (DKA) [35]. Our findings indicate that the pooled percentage of individuals with DKA was 6.67%, consistent with the findings of DECLARE–TIMI, CANVAS, and STICH Protocol [31,35,38].

In our investigation, glycosuria is found to be one of the main side effects of employing SGLT2-i and also raised the chance of infection, primarily genitourinary tract infections. Three studies were examined regarding the driveline infection rate, and the pooled percentage of those with driveline infection was 9%. DECLARE–TIMI, the EMPA-REG OUTCOME trial (2015), and the EMPA-REG OUTCOME (2018) all reported an increased risk of SGLT2-i induced infection [25,35,39].

Although post-marketing data has documented cases of acute renal damage, we found that the combined percentage of AKI cases was 2% in three investigations and that the EMPA-REG OUTCOME study and the CANVAS PROGRAMME showed no increased risk [25,31].

Our pooled data from BNP and GFR showed non-statistical significance despite causing improvement. Regarding BNP and GFR, the type of SGLT2-i used and the difference in baseline characteristics in the population can cause a different effect, which explains the variability.

This meta-analysis will help clinicians and researchers find the optimal dose and duration of SGLT2-i that will help patients with LVADs improve their outcomes. Also, it opens the door for future trials to see if SGLT2-i significantly helps patients with LVADs.

Our systematic review of SGLT2-i in LVAD patients is considered the first one investigating the effect of this drug among LVAD patients and leads to further understanding of this drug despite the lack of data on SGLT2-i among this population. It emphasized that SGLT2-i could be a promising drug for management and lead to improvement in the clinical parameters in LVAD patients.

### Limitations

Several limitations were encountered while conducting this systematic review. The most important one is the low number of participants and few published studies about SGLT2-i in patients using LVADs, which included only three published articles and seven published conference abstracts. Another limitation is the nature and design of the published papers and the lack of RCTs investigating SGLT2-i in LVAD patients, which increases the need to establish further trials among this population.

## 5. Conclusions

SGLT2-i helped improve BNP and HbA1c in patients using LVADs and showed a good safety profile among the participants. Future RCTs should be established to include large numbers of LVAD patients to investigate the safety and efficacy of SGLT2-i across different parameters and measure the effect of SGLT2-i over time among this population.

## Figures and Tables

**Table 1:** Baseline information of patients with LVADs using SGLT2-i.

Study	N	Age, Years	Male	Duration of Support (Duration of LVAD)	INTERMACS Level				
					1	2	3	4	5
Cagliostro et al., 2022 [7]	34	56.1 ± 10.6	27 (79.4%)	-	5 (15.6%)	5 (15.6%)	19 (59.4%)	3 (9.4%)	0 (0%)
Chavali et al., 2023 [5]	16	52.25 (46.71–57.93)	15 (93.75%)	212 (133–324) days	6 (37.5%)	3 (18.75%)	3 (18.75%)	4 (25%)	0 (0%)
Moady et al., 2023 [6]	20	64.7 ± 12.2	15 (75%)	-	2 (10%)	5 (25%)	8 (40%)	5 (25%)	0 (0%)
Fardman et al., 2024 [21]	29	62 ± 6.7	25 (86.2%)	-	2 (6.9%)	9 (31%)	12 (41.4%)	6 (20.7%)	0 (0%)
**Conference abstracts**									
Hambright et al., 2022 [16]	21	-	-	-	-	-	-	-	-
Acosta et al., 2022 [14]	15		-	-	-	-	-	-	-
Yaranov et al., 2022 [17]	9	54	36 (67%)	-	-	-	-	-	-
Chavali et al., 2022 [15]	12	-	-	-	-	-	-	-	-
Byczkowska et al., 2023 [18]	51	60 ± 3.5	51 (100%)	-	-	-	-	-	-
Lanham et al., 2023 [19]	1	42	1 (100%)	-	-	-	-	-	-
Al Ali et al., 2024 [20]	20	59 ± 11	17 (85%)	-	-	-	-	-	-

Data are presented as numbers (percentages); Mean ± SD; Median (IQR). Abbreviations: INTERMACS: Interagency Registry for Mechanically Assisted Circulatory Support; IQR: Interquartile Range; LVAD: Left Ventricular Assist Device; SD: Standard Deviation; SGLT2-i: Sodium-Glucose Co-Transporter-2 Inhibitors.

**Table 2:** Risk factors, types of SGLT2-i and LVADs among participants.

Study	Risk Factors					SGTL2-i				Types of LVAD		
	HTN	DM	MI	CABG	CKD	Cana	Empa	Dapa	Ertu	HVAD	HM2	HM3
Cagliostro et al., 2022 [7]	-	34 (100%)	10 (29.4%)	4 (11.8%)	16 (47.1%)	2 (5.9%)	22 (64.7%)	10 (29.4%)	0 (0%)	9 (26.5%)	9 (26.5%)	9 (26.5%)
Chavali et al., 2023 [5]	-	2 (12.50%)	-	-	-	-	-	-	-	-	-	-
Moady et al., 2023 [6]	15 (75%)	20 (100%)	-	-	-	-	14 (70.0%)	6 (30.0%)	-	-	-	20 (100%)
Fardman et al., 2024 [21]	-	-	-	-	-	-	23 (79%)	6 (21%)	-	-	-	29 (100%)
**Conference abstracts**												
Hambright et al., 2022 [16]	-	-	-	-	-	-	-	-	-	-	-	-
Acosta et al., 2022 [14]	-	10 (66.67%)	-	-	-	-	-	-	-	-	-	-
Yaranov et al., 2022 [17]	-	-	-	-	-	-	Received	Received	-	-	-	-
Chavali et al., 2022 [15]	-	-	-	-	-	-	-	-	-	-	-	-
Byczkowska et al., 2023 [18]	-	33%	-	-	-	-	-	-	-	-	-	-
Lanham et al., 2023 [19]	-	1 (100%)	-	-	-	-	1 (100%)	-	-	-	-	-
Al Ali et al., 2024 [20]	-	-	-	-	-	1 (5%)	6 (30%)	12 (60%)	1 (5%)	0 (0%)	0 (0%)	20 (100%)

Data are presented as numbers (percentages). Abbreviations: CABG: Coronary Artery Bypass Graft; Cana: Canagliflozin; Dapa: Dapagliflozin; Ertu: Ertugliflozin; Empa: Empagliflozin; HM2: Heartmate 2; HM3: Heartmate 3; HVAD: Heartware Ventricular Assist Device; LVAD: Left Ventricular Assist Device; SGLT2-i: Sodium-Glucose Co-Transporter-2 Inhibitors.

**Table 3:** Summary of included studies of patients with LVADs using SGLT2-i.

Study	Study Design	Follow-Up	Dose	Duration of SGLT2-i (Days)	Device Strategy
Cagliostro et al., 2022 [7]	Retrospective study	30 days, 60 days, and 180 days	-	-	BTT: 11 (35.5%) DT: 20 (64.5%)
Chavali et al., 2023 [5]	Retrospective study	-	-	101.5 (37.5–190.8)	BTT
Moady et al., 2023 [6]	Observational study	6 months	10 mg once daily	-	-
Fardman et al., 2024 [21]	Retrospective study	6 months	10 mg once daily	-	-
**Conference abstracts**					
Lanham et al., 2023 [19]	Case report	-	-	-	-
Acosta et al., 2022 [14]	Retrospective study	-	-	-	-
Yaranov et al., 2022 [17]	-	-	-	-	-
Chavali et al., 2022 [15]	-	-	-	-	BTT
Hambright et al., 2022 [16]	Retrospective study	3 months	-	-	-
Byczkowska et al., 2023 [18]	-	3 months 1 year	-	-	-
Al Ali et al., 2024 [20]	Retrospective study	-	-	-	-

Data are presented as numbers (percentages), median (IQR). Abbreviations: BTT: Bridge-To-Transplant; DT: Destination Therapy; IQR: Interquartile Range.

**Table 4:** Summary of the effect of SGLT2-i on echocardiography outcomes in patients with LVADs.

Study	EF	CIn	LVEDD	LVESD	BNP	MAP	MPAP	mPCWP	PVR	TPG	RAP	SVR
Cagliostro et al., 2022 [7]	-	-	-	-	↔	-	-	-	-	-	-	-
Chavali et al., 2023 [5]	-	↔	-	-	-	↔	-	↓	↓	↓	↔	↔
Moady et al., 2023 [6]	-	-	-	-	-	↔	-	-	-	-	-	-
Fardman et al., 2024 [21]	↔	-	↔	↔	-	-	↔	-	-	-	-	-
**Conference abstracts**												
Lanham et al., 2023 [19]	-	-	-	-	-	-	-	-	-	-	-	-
Acosta et al., 2022 [14]	-	-	-	-	↓	-	-	-	-	-	-	-
Yaranov et al., 2022 [17]	-	-	-	-	-	-	-	-	-	-	-	-
Chavali et al., 2022 [15]	-	-	-	-	-	-	-	-	-	-	-	-
Hambright et al., 2022 [16]	-	-	-	-	-	-	-	-	-	-	-	-
Byczkowska et al., 2023 [18]	-	-	-	-	↓	-	-	-	-	-	-	-
Al Ali et al., 2024 [20]	↔	-	-	-	↔	-	-	-	-	-	-	-

Abbreviations: BNP: Brain Natriuretic Peptide; CIn: Cardiac Index; EF: Ejection Fraction; LVEDD: Left Ventricular End-Diastolic Diameter; LVESD: Left Ventricular End-Systolic Diameter; MAP: Mean Arterial Pressure; MPAP: Mean Pulmonary Arterial Pressure; mPCWP: Mean Pulmonary Capillary Wedge Pressure; PVR: Pulmonary Vascular Resistance; RAP: Right Atrial Pressure; SVR: Systemic Vascular Resistance; TPG: Transpulmonary Pressure Gradient.

## Data Availability

Not applicable.

## Supplementary Materials

The following supporting information can be downloaded at: https://www.mdpi.com/article/10.3390/jcm13237418/s1. Supplementary Table S1: Search strategy for the studies in databases; Supplementary Table S2: Quality assessment of included studies using adapted NOS and adapted NOS; Supplementary Table S3: Summary of the effect of SGLT2-i on other parameters in patients with LVAD.

## Abbreviations

6MWT	6-Minute Walk Test
AKI	Acute Kidney Injury
BMI	Body Mass Index
BNP	B-Type Natriuretic Peptide
BTT	Bridge to Transplant
BUN	Blood Urea Nitrogen
CABG	Coronary Artery Bypass Graft
Cana	Canagliflozin
CI	Confidence Interval
Cin	Cardiac Index
Cr	Creatinine
Dapa	Dapagliflozin
DKA	Diabetic Ketoacidosis
DM	Diabetes Mellitus
DT	Destination Therapy
EF	Ejection Fraction
Empa	Empagliflozin
Ertu	Ertugliflozin
GFR	Glomerular Filtration Rate
HbA1c	Hemoglobin A1c
HF	Heart Failure
HFpEF	Heart Failure with Preserved Ejection Fraction
HFrEF	Heart Failure with Reduced Ejection Fraction
HM2	Heartmate 2
HM3	Heartmate 3
HVAD	Heartware Ventricular Assist Device
INTERMACS	Interagency Registry for Mechanically Assisted Circulatory Support
IQR	Interquartile Range
KCCQ	Kansas City Cardiomyopathy Questionnaire
LVAD	Left Ventricular Assist Device
LVAS	Left Ventricular Assist System
LVEDD	Left Ventricular End-Diastolic Diameter
LVESD	Left Ventricular End-Systolic Diameter
MAP	Mean Arterial Pressure
MPAP	Mean Pulmonary Artery Pressure
mPCWP	Mean Pulmonary Capillary Wedge Pressure
NOS	Newcastle–Ottawa Scale
NYHA class	New York Heart Association Class
PAP	Pulmonary Artery Pressure
PRISMA	Preferred Reporting Items for Systematic Reviews And Meta-Analyses
PVR	Pulmonary Vascular Resistance
RAAB	Renin Angiotensin Aldosterone Blockers
RAP	Right Atrial Pressure
RCTs	Randomized Controlled Clinical Trials
RVAD	Right Ventricular Assist Device
SBP	Systolic Blood Pressure
SD	Standard Deviation
SE	Standard error
SGLT2-i	Sodium-Glucose Co-Transporter-2 Inhibitors
SVR	Systemic Vascular Resistance
T2DM	Type 2 Diabetes Mellitus
TPG	Transpulmonary Pressure Gradient
