# Supplementary material for: A Systematic Review and Meta-Analysis of the Efficacy and Safety of Sodium-Glucose Cotransporter-2 Inhibitor in Patients Using Left Ventricular Assist Devices
Source: J Clin Med. 2024 Dec 5;13(23):7418. doi: 10.3390/jcm13237418 (PMC11641912; doi:10.3390/jcm13237418)
Supplement: Supplementary file 1 [file jcm-13-07418-s001.zip › jcm-3300066-supplementary.pdf]

Supplementary file:

1. **Supplementary Table S1**: Search strategy of the studies in databases.
2. **Supplementary Table S2:** Quality assessment of included studies using adapted NOS and adapted NOS
3. **Supplementary Table S3.** Summary of the effect of SGLT2-i across on other parameters in patients with LVAD.

Supplementary Table S1: Search strategy of the studies in databases.

PubMed	((((((((((((((((((((((((((SGLT2) OR (SGLT2 inhibitor)) OR (SGLT-2 inhibitor)) OR (sodium glucose cotransporter 2 Inhibitor)) OR (Sodium Glucose Transporter 2 Inhibitors)) OR (sodium glucose co-transporter 2)) OR (sodium glucose co-transporter 2 inhibitor)) OR (sodium glucose cotransporter 2)) OR (SGLT-2i)) OR (gliflozin)) OR (Gliflozins)) OR (canagliflozin)) OR (empagliflozin)) OR (dapagliflozin)) OR (Ipragliflozin)) OR (tofogliflozin)) OR (luseogliflozin)) OR (ertugliflozin)) OR (sotagliflozin)) OR (remogliflozin)) OR (Invokana)) OR (Farxiga)) OR (Jardiance)) OR (Suglat)) OR (Apleway)) OR (Deberza)) AND (((((((((((((((((((left ventricular assist device) OR (lvad)) OR (heartmate)) OR (heartware)) OR (heart pump)) OR (jarvik-2000)) OR (thoratec)) OR (ventricular assist device)) OR (vad)) OR (Mechanical Circulatory Support)) OR (mcs)) OR (biventricular assist device)) OR (bivad)) OR (Heart Assist Device)) OR (novacor)) OR (left ventricular assist system)) OR (LVAS)) OR (RVAD)) OR (right ventricular assist device))	54
Scopus	(TITLE-ABS-KEY ((sglt OR "sglt inhibitor" OR "SGLT-2 inhibitor" OR "sodium glucoase coatransporter 2 inhibitor" OR "sodium glucoase transporter 2 inhibitors" OR "sodium glucoase coa-transporter 2" OR " sodium glucoase coa-transporter 2 inhibitor" OR " sodium glucoase coatransporter 2" OR "SGLT-2i" OR albiflorin OR gliotoxins OR sanglifehrin OR parviflorin OR parviflorin OR parviflorin OR paeoniflorin OR paeoniflorin OR prulifloxacin OR albiflorin OR hemoglobin OR invoking OR farina OR variance OR sugar OR alleyway OR debra)) AND TITLE-ABS-KEY (("left ventricular assist device" OR lead OR heartwater OR heartwater OR "heart pump" OR jarvik-2000 OR thoriated OR "ventricular assist device" OR vad OR "Mechanical Circulatory Support" OR mcs OR	42

	"biventricular assist device" OR ivad OR heart AND assist AND device OR vacor OR "left ventricular assist system" OR lavas OR road OR right AND ventricular AND assist AND device)))	
Web Of Science	(SGLT2 OR "SGLT2 inhibitor" OR "SGLT-2 inhibitor" OR "sodium glucose cotransporter 2 Inhibitor" OR "Sodium Glucose Transporter 2 Inhibitors" OR "sodium glucose co-transporter 2" OR " sodium glucose co-transporter 2 inhibitor" OR " sodium glucose cotransporter 2" OR "SGLT-2i" OR gliflozine OR gliflozine OR canagliflozin OR empagliflozin OR dapagliflozin OR Ipragliflozin OR tofogliflozin OR luseogliflozin OR ertugliflozin OR sotagliflozin OR remogliflozin OR invoking OR forxiga OR jardine OR suglet OR ayleway OR deberia) (All Fields) and ("left ventricular assist device" OR lvad OR heartmate OR heartware OR "heart pump" OR jarvik-2000 OR thoratic OR "ventricular assist device" OR vad OR "Mechanical Circulatory Support" OR mcs OR "biventricular assist device" OR bilad OR "Heart Assist Device" OR novaco OR "left ventricular assist system" OR lvad OR road OR "right ventricular assist device") (All Fields)	35
Cochrane Library	(SGLT2 OR "SGLT2 inhibitor" OR "SGLT-2 inhibitor" OR "sodium glucose cotransporter 2 Inhibitor" OR "Sodium Glucose Transporter 2 Inhibitors" OR "sodium glucose co-transporter 2" OR " sodium glucose co-transporter 2 inhibitor" OR " sodium glucose cotransporter 2" OR "SGLT-2i" OR gliflozin OR Gliflozins OR canagliflozin OR empagliflozin OR dapagliflozin OR Ipragliflozin OR tofogliflozin OR luseogliflozin OR ertugliflozin OR sotagliflozin OR remogliflozin OR Invokana OR Farxiga OR Jardiance OR Suglat OR Apleway OR Deberza)<br>AND<br>("left ventricular assist device" OR lvad OR heartmate OR heartware OR "heart pump" OR jarvik-2000 OR thoratec OR "ventricular assist device" OR vad OR "Mechanical Circulatory Support" OR mcs OR "biventricular assist device" OR bivad OR "Heart Assist Device" OR	3

	novacor OR "left ventricular assist system" OR LVAS OR RVAD OR “right ventricular assist device”)	
Embase	(sglt2 OR 'sglt2 inhibitor'/exp OR 'sglt2 inhibitor' OR 'sglt-2 inhibitor' OR 'sodium glucose cotransporter 2 inhibitor'/exp OR 'sodium glucose cotransporter 2 inhibitor' OR 'sodium glucose transporter 2 inhibitors'/exp OR 'sodium glucose transporter 2 inhibitors' OR 'sodium glucose co-transporter 2' OR 'sodium glucose co-transporter 2 inhibitor'/exp OR 'sodium glucose co-transporter 2 inhibitor' OR 'sodium glucose cotransporter 2'/exp OR 'sodium glucose cotransporter 2' OR 'sglt-2i' OR 'gliflozin'/exp OR gliflozin OR 'gliflozins'/exp OR gliflozins OR 'canagliflozin'/exp OR canagliflozin OR 'empagliflozin'/exp OR empagliflozin OR 'dapagliflozin'/exp OR dapagliflozin OR 'ipragliflozin'/exp OR ipragliflozin OR 'tofogliflozin'/exp OR tofogliflozin OR 'luseogliflozin'/exp OR luseogliflozin OR 'ertugliflozin'/exp OR ertugliflozin OR 'sotagliflozin'/exp OR sotagliflozin OR 'remogliflozin'/exp OR remogliflozin OR 'invokana'/exp OR invokana OR 'farxiga'/exp OR farxiga OR 'jardiance'/exp OR jardiance OR 'suglat'/exp OR suglat OR 'apleway'/exp OR apleway OR 'deberza'/exp OR deberza) AND ('left ventricular assist device'/exp OR 'left ventricular assist device' OR 'lvad'/exp OR lvad OR 'heartmate'/exp OR heartmate OR 'heartware'/exp OR heartware OR 'heart pump'/exp OR 'heart pump' OR 'jarvik 2000'/exp OR 'jarvik 2000' OR 'thoratec'/exp OR thoratec OR 'ventricular assist device'/exp OR 'ventricular assist device' OR vad OR 'mechanical circulatory support'/exp OR 'mechanical circulatory support' OR mcs OR 'biventricular assist device'/exp OR 'biventricular assist device' OR 'bivad'/exp OR bivad OR 'heart assist	244

	device'/exp OR 'heart assist device' OR 'novacor'/exp OR novacor OR 'left ventricular assist system' OR lvas OR rvad OR 'right ventricular assist device'/exp OR 'right ventricular assist device')	
**Total**		378

Supplementary Table S2: Quality assessment of included studies using adapted NOS and adapted NOS

Study	Selection	Comparability	Outcome	Total score
Cagliostro et al. 2022 [7]	3	-	3	6
Chavali et al. 2023 [5]	3	2	3	8
Moady et al. 2023 [6]	3	-	2	5
Fardman et al. 2024 [21]	3	-	3	6

Supplementary Table S3. Summary of the effect of SGLT2-i across on other parameters in patients with LVAD.

Study	NYHA class	Cr	GFR	Sodium	Potassium	Bicarbonate	SBP	BUN	BMI	HbA1c	Diuretic dose	Weight	6MWT	KCCQ
Cagliostro et al. 2022[7]	-	↔	↔	-	↔	↔	↔	↓	↔	↔	↔	-	-	-
Chavali et al. 2023[5]	-	-	↔	-	-	-	-	-	-	-	-	↔	-	-
Moady et al. 2023[6]	-	-	↔	-	-	-	-	-	-	↓	-	-	-	-
Fardman et al. 2024 [21]	-	↔	↔	↔	↑	-	↔	-	-	-	↓	↓	-	-
**Conference abstracts**														
Lanham et al. 2023 [19]	-	-	-	-	-	-	-	-	-	-	-	-	-	-
Acosta et al. 2022 [14]	-	-	↔	-	-	-	-	-	-	↔	-	-	-	-
Yaranov et al. 2022 [17]	-	-	-	-	-	-	-	-	-	-	↓	-	-	-
Chavali et al. 2022 [15]	-	-	↔	-	-	-	-	-	-	-	-	-	-	-
Hambright et al. 2022 [16]	-	-	-	-	-	-	-	-	-	-	↓	-	-	-
Byczkowska et al. 2023 [18]	↑	-	-	-	-	-	-	-	-	-	-	-	↑	-
Al Ali et al. 2024 [20]	-	-	↔	-	-	-	-	-	-	↔	-	-	-	↑

Abbreviations: 6MWT: 6-Minute Walk Test; BMI: Body Mass Index; BUN: Blood Urea Nitrogen; Cr: Creatinine; GFR: Glomerular Filtration Rate; HbA1c: Hemoglobin A1c; KCCQ: Kansas City Cardiomyopathy Questionnaire; NYHA class: New York Heart Association Class; SBP: Systolic Blood Pressure
